# Cyclin D1 overexpression is a negative predictive factor for tamoxifen response in postmenopausal breast cancer patients

**DOI:** 10.1038/sj.bjc.6601831

**Published:** 2004-04-27

**Authors:** M Stendahl, Å Kronblad, L Rydén, S Emdin, N O Bengtsson, G Landberg

**Affiliations:** 1 Division of Pathology, Department of Laboratory Medicine, Lund University, Malmö University Hospital, S-20502 Malmö, Sweden; 2 Department of Surgery, Umeå University, Umeå, Sweden; 3 Department of Oncology, Umeå University, Umeå, Sweden

**Keywords:** cyclin D1, breast cancer, oestrogen receptor, tamoxifen, predictive factors

## Abstract

Antioestrogen treatment by tamoxifen is a well-established adjuvant therapy for oestrogen receptor-alpha (ER*α*) positive breast cancer. Despite ER*α* expression some tumours do not respond to tamoxifen and we therefore delineated the potential link between the cell cycle regulator and ER*α* co-factor, cyclin D1, and tamoxifen response in a material of 167 postmenopausal breast cancers arranged in a tissue array. The patients had been randomised to 2 years of tamoxifen treatment or no treatment and the median follow-up time was 18 years. Interestingly in the 55 strongly ER*α* positive samples with moderate or low cyclin D1 levels, patients responded to tamoxifen treatment whereas the 46 patients with highly ER*α* positive and cyclin D1 overexpressing tumours did not show any difference in survival between tamoxifen and no treatment. Survival in untreated patients with cyclin D1 high tumours was slightly better than for patients with cyclin D1 low/moderate tumours. However, there was a clearly increased risk of death in the cyclin D1 high group compared to an age-matched control population. Our results suggest that cyclin D1 overexpression predicts for tamoxifen treatment resistance in breast cancer, which is line with recent experimental data using breast cancer cell lines and overexpression systems.

Breast cancer is a highly heterogeneous disease that ideally should be subcategorised according to genetic defects potentially reflecting prognostic and predictive information in order to assure optimal and individualised treatment for patients (Sorlie *et al*, 2001). Adjuvant treatment with antioestrogens like tamoxifen is one of the most important treatment strategies used for breast cancer, saving many lives. The presence of oestrogen receptor-alpha (ER*α*) in tumour cells is essential for tamoxifen response, and together with the progesterone receptor it serves as a predictive factor for tamoxifen response in clinical practice (EBCTC, 1998). Despite ER*α* expression some tumours do not respond or develop resistance to tamoxifen treatment, suggesting that the presence of ER*α* is not the only factor influencing tamoxifen response (Ali and Coombes, 2002). Even though the rationale for treatment failure is not fully comprehended, co-factors to the ER*α* such as cyclin D1 might be implicated in the process, theoretically blocking tamoxifen response. Cyclin D1 is a cell cycle regulating protein with potential dual roles and in addition to activating cdk 4/6 and sequestering of cdk-inhibitors in the G1/S transition (Zhou *et al*, 2001), the protein has cdk-independent functions and can act as a co-factor for ER*α* independent of ligand (Neuman *et al*, 1997; Zwijsen *et al*, 1997, 1998; McMahon *et al*, 1999; Lamb *et al*, 2000). It has further been shown that cyclin D1 overexpression can affect tamoxifen response in breast cancer cell lines (Hui *et al*, 2002), but contradictory results have nevertheless been published regarding this potentially important feature for cyclin D1 (Pacilio *et al*, 1998; Bindels *et al*, 2002). No randomised clinical studies detailing cyclin D1 in ER*α* positive breast cancer with regards to tamoxifen response have been reported. Cyclin D1 knockout mice show a marked defect in breast epithelium development during pregnancy (Sicinski *et al*, 1995) and tissue-specific overexpression of cyclin D1 leads to mammary hyperplasia and adenocarcinoma formation in mice models (Wang *et al*, 1994), supporting the relevance for cyclin D1 in breast cancer development.

Besides a potential role for cyclin D1 in ER*α* response, cyclin D1 has also been linked to prognostic information in breast cancer. Cyclin D1 gene amplification in sporadic tumours as well as cyclin D1 RNA expression in ER*α* positive breast cancer has been correlated to poor prognosis (Kenny *et al*, 1999; Bieche *et al*, 2002; Naidu *et al*, 2002). When studying protein levels, cyclin D1 overexpression has in contrast been associated with better outcome (Gillett *et al*, 1996; Hwang *et al*, 2003). Furthermore, cyclin D1 overexpression has been closely linked to ER*α* positivity (Michalides *et al*, 1996; van Diest *et al*, 1997). In order to better clarify the role of cyclin D1 in breast cancer we analysed a material of randomised postmenopausal breast cancer patients with long follow-up. Interestingly, cyclin D1 overexpression was associated with tamoxifen resistance despite the presence of ER*α*. Tumours overexpressing cyclin D1 were further associated with a slightly improved survival, and in summary we show that randomised treatment trials including an untreated control arm are necessary in order to differ predictive and prognostic information.

## MATERIALS AND METHODS

### Study design

Between 1980 and 1987, 248 patients with primary breast cancer were enrolled in a clinical trial (EBCTC, 1998) in the Northern Health Region of Sweden and randomised to adjuvant tamoxifen treatment (40 mg daily), *n*=123, for 2 years *vs* control, *n*=125. The aim of the study was to compare the effect of tamoxifen *vs* no treatment on overall survival. The inclusion criteria were postmenopausal patients >55 years of age operated with modified radical mastectomy with curative intent. Postoperative radiotherapy was given to all patients with lymph node metastases. Hormone receptor status was not evaluated at time of diagnosis.

The median age at diagnosis was 66.5 years (55–75 years), the median tumour size was 25 mm (>0–76 mm) and 159 node-negative patients and 86 node-positive patients were encountered. According to TNM-classification 65 patients with stage I disease, 175 patients with stage II and five patients with stage III disease, were included in survival analyses. Three patients could not be classified according to TNM.

The median follow-up time was 18 years (range 15–22 years) for surviving patients. At 10-years of follow-up 72 patients (58%) were alive in the control group and 76 (62%) in the tamoxifen group. In the control group 53 (42%) patients had died and in the tamoxifen group 47 (38%) patients had died. At the last follow-up 34% of the patients in the control group were alive, whereas 66% had died. In the tamoxifen group 34% were alive and 66% had died. All clinicopathological data together with clinical outcome are summarised according to treatment arm in Table 1

**Table 1:** Patient and tumour characteristics according to treatment

**Variable**	**Tamoxifen treated patients (*n*=123)**	**Control (*n*=125)**
*Age (years) median*	66.2 (54.9–74.7)	66.8 (55.2–74.9)
		
*Age (number)*		
>Median 66 years	60	65
<Median 66 years	63	60
		
*Tumour size*		
Median (mm)	25.0 (3–55)	24.5 (>0–76)
Size not known	2	0
		
*Node status*		
Node−	80	79
Node+	41	45
Not known	2	1
		
*Stage*		
I	30	35
II	90	85
III	1	4
Not known	2	1
		
*ERα fraction*		
0–10%	19	29
10–90%	14	12
>90%	53	55
Not known	37 (30%)	29 (23%)
		
*PR fraction*		
0–10%	47	50
10–90%	20	22
>90%	17	18
Not known	39 (32%)	35 (28%)
		
*Cyclin D1 fraction*		
0–10%	21	21
10–90%	53	53
>90%	9	15
Not known	40 (33%)	36 (29%)
		
*Cyclin D1 intensity*		
Negative	0	1
Low	4	5
Moderate	50	49
High	29	34
Not known	40 (33%)	36 (29%)
		
*10-year FU*		
Alive	76	72
Dead	47	53
		
*Maximum FU*		
Alive	42	42
Dead	81	83

.

### Tissue array and immunohistochemistry

Representative parts of the tumours were assembled in a tissue array using a manual tissue arrayer (Beecher Instruments Micro-array Technology, Woodland, MD, USA). For immunohistochemistry, paraffin sections of 4 *μ*m were de-paraffinised using xylen and rehydrated using descending concentrations of ethanol according to standard protocol. Antigen retrieval was achieved using 10 mM citrate buffer at pH 6.0 and microwave treatment, before incubations with antibodies to the ER*α* (M7047, 1 : 200, Dako A/S, Glostrup, Denmark) and cyclin D1 (M7155, 1 : 100, Dako). All stainings were performed in a Dako Techmate 500 (Dako). The specificity of the antibodies was confirmed by comparing the immunohistochemistry stainings of cell lines with Western blot reactivity (data not shown).

### Analytical procedures

The randomised study included both ER*α* positive and ER*α* negative breast cancers and we therefore initially delineated the fraction of ER*α* positive tumour cells in four groups (0, <10, 10–90 and >90%) for each patient. ER*α* was scored as positive if >10% and the subfraction of >90% was used for the analyses of a homogenous group of potentially highly tamoxifen responsive tumours. Cyclin D1 protein was monitored by two investigators scoring the tissue array samples according to intensity and fraction of the nuclear staining. Fraction was scored according to the same criteria as ER*α* and intensity was subjectively divided into four groups (negative, low, moderate, high), see Table 1. By using tissue array, staining variations between samples were minimised and positive samples could be used as supplements for internal controls. Concordance between the investigators was high and in the few cases of differing results, biopsies were re-evaluated and discussed to reach consensus. Relevant tissue array biopsies for ER*α*/cyclin D1 analyses were obtained for 167 tumours. In total, 56 biopsies contained insufficient tumour cells or did not sustain the staining process. A total of 44 tumours were ER*α* negative (0–10% positive fraction), 22 had 10–90% and 101 >90% ER*α* positive cells (Table 2

**Table 2:** Cyclin D1 intensity and ER*α* fraction analysis

	**ER*α* fraction**		
**Cyclin D1 intensity**	**0–10%**	**10–90%**	**>90%**
Negative	1	0	0
Low	3	1	5
Moderate	35	13	50
High	5	8	46
Total (*n*=167)	44	22	101

). For the survival analysis both intensity and fraction of cyclin D1 were used (Tables 2 and 3

**Table 3:** Cyclin D1 fraction and ER*α* fraction analysis

	**ER*α* fraction**		
**Cyclin D1 fraction**	**0–10%**	**10–90%**	**>90%**
0–10%	28	5	7
10–90%	16	15	74
>90%	0	2	20
Total (*n*=167)	44	22	101

).

### Statistical analysis

Kaplan–Meier's plot and log rank test were used when illustrating and calculating survival. All calculations were performed in SPSS version 11.0. An age-matched survival estimate for a control population in southern Sweden was obtained from the Cancer Registry in Lund, Sweden.

## RESULTS AND DISCUSSION

**Figure 1:**
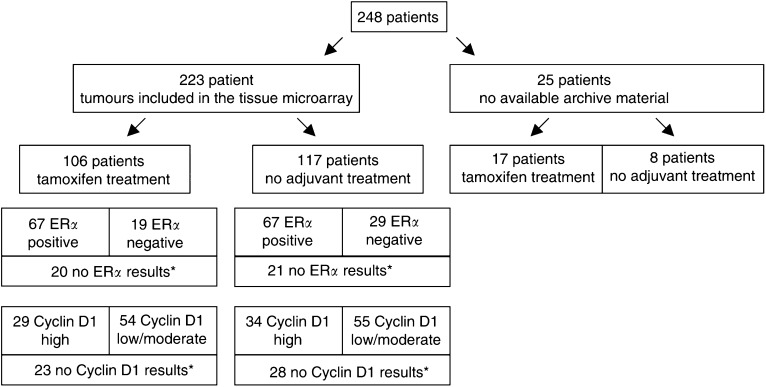
Illustration of the patient material, randomisation and distribution of the analyses results. ER*α* positivity is >10% positive cells and the cyclin D1 results are based on intensity determinations. ^*^No evaluable tumour cells in the array or the tumour did not sustain the staining process.

Of the 248 patients included in the randomised treatment trial, formalin-fixed material was available for 223 tumours and 106 of these patients were randomised to tamoxifen treatment and 117 to observation, as described in Figure 1

. Of the 182 breast cancer samples that were possible to evaluate for ER*α* in the array, 74% were classified as positive, which is in agreement with other reports including postmenopausal patients (Elwood and Godolphin, 1980). In total, 59% of the tumours showed >90% ER*α* positive cells, which is slightly higher than expected compared to ELISA-based analyses (Thorpe *et al*, 1993). A summary of the 172 samples that were possible to evaluate for cyclin D1, including intensity and fraction is presented in Table 1. The cyclin D1 and ER*α* analyses combined are presented in Tables 2 and 3. The fraction of cyclin D1 positive tumour cells and the cyclin D1 intensity determinations correlated significantly (*P*<0.0001, *r*=0.463) and were both used for the following survival analysis regarding tamoxifen response.

**Figure 2:**
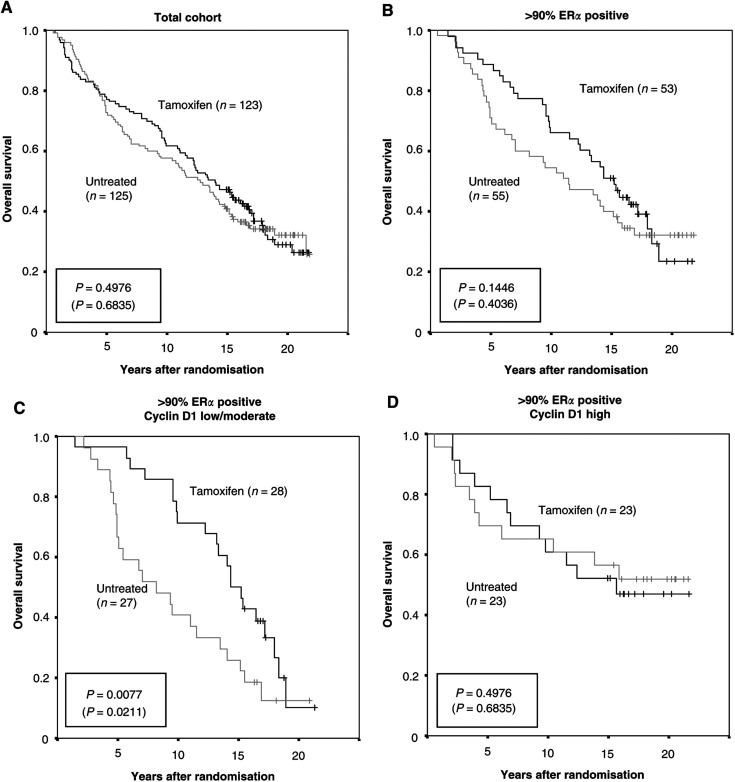
Kaplan–Meier plots for overall survival indicating randomisation to 2 years of tamoxifen treatment contra no treatment. (**A**) Overview of the entire cohort including both ER*α* positive and negative tumours. (**B**) The subgroup of 101 >90% ER*α* positive breast cancer samples. (**C**, **D**) Overall survival for patients having tumours with >90% ER*α* positivity in relation to cyclin D1 intensity (low/moderate or high expression). *P*-values are presented in a box with values at 10 years of follow-up (22 years of follow-up in parenthesis).

In the total cohort consisting of both ER*α* positive and negative tumours, tamoxifen treatment did not improve prognosis (Figure 2A

). Patients with tumours lacking or expressing low ER*α* did, as expected, not show any significant difference in survival with tamoxifen, whereas there was a slightly improved survival for patients with tumours having >10% ER*α* positive cells (*P*=0.4055 after 10 years of follow-up and *P*=0.6000 after 22 years). The 108 tumours with >90% ER*α* positive cells showed nevertheless a more pronounced response to tamoxifen treatment as illustrated in Figure 2B, supporting that there was an increased effect of adjuvant tamoxifen treatment in ER*α* high tumours compared to tumours with moderately elevated ER*α* levels. In order to delineate the effect of cyclin D1 expression in relation to tamoxifen response, overall survival in patients with ER*α* positive tumours was stratified according to the fraction and intensity of cyclin D1 expression and potential differences between tamoxifen and no treatment were evaluated. The fraction of cyclin D1 positive cells did not seem to influence the effect of tamoxifen treatment and survival curves with an apparent tamoxifen benefit were comparable in the cyclin D1 fraction subgroups (data not shown). Similar results were obtained both for the entire ER*α* positive cohort and the subgroup of >90% ER*α* positive tumours. In contrast, the cyclin D1 intensity analyses showed substantial differences in tamoxifen response. A total of 45% of the 101 ER*α* high tumours had high cyclin D1 intensity staining whereas 50% had moderate and 5% were cyclin D1 low. By this definition, around half of the ER*α* positive tumours overexpressed cyclin D1, which is in line with earlier reports (Gillett *et al*, 1996; Michalides *et al*, 1996). When analysing all ER*α* positive tumours separating moderate/low cyclin D1 from high cyclin D1 tumours, a clear distinction in tamoxifen response was observed. In the group with lower cyclin D1, there was a difference in survival between tamoxifen and no treatment in the entire ER*α* positive cohort (*P*=0.0509 at 10 years and *P*=0.0927 at 22 years). When analysing the 101 tumours with >90% ER*α* positive cells this effect was even more distinct (*P*=0.0077 at 10 years, *P*=0.0211 at 22 years), as illustrated in Figure 2C. Surprisingly, the difference between tamoxifen and no treatment was completely eliminated for tumours with high cyclin D1 (Figure 2D). This suggests that overexpression of cyclin D1 was linked to tamoxifen treatment resistance despite high and homogenous ER*α* content.

**Figure 3:**
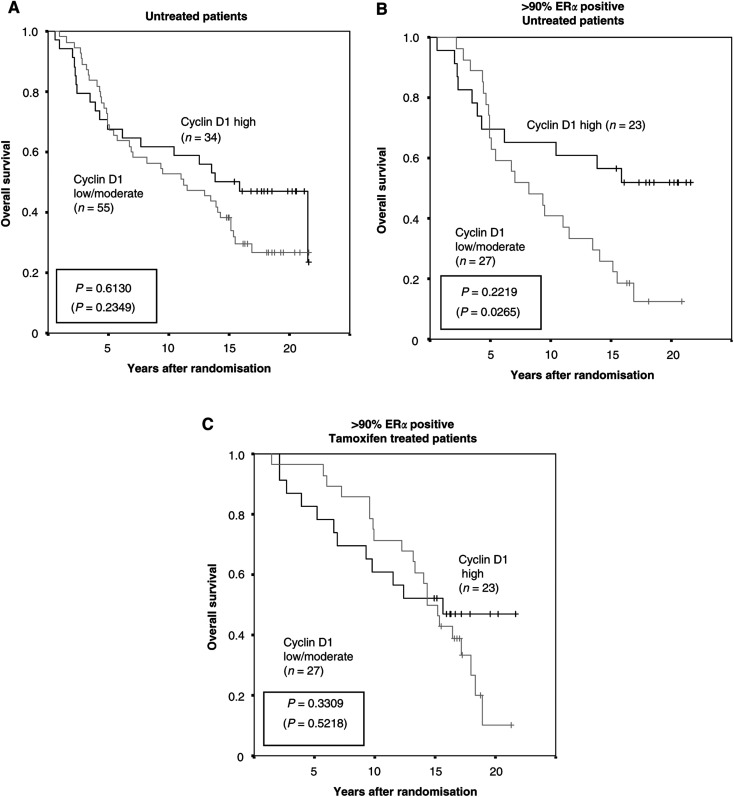
Overall survival in relation to cyclin D1 intensity (low/moderate and high) in (**A**) all untreated patients, (**B**) untreated patients with >90% ER*α* positive cells and (**C**) tamoxifen treated patients with >90% ER*α* positive cells. *P*-values are presented in a box with values at 10 years of follow-up (22 years of follow-up in parenthesis).

Regarding survival and cyclin D1, the untreated control patients could be used to evaluate true prognostic information without the interference of treatment differences. The fraction of cyclin D1 positive cells was not associated with any prognostic data whereas there was a difference in survival for cyclin D1 groups based on intensity determination, as illustrated in Figure 3

. For all untreated patients there was a mortality rate of 56 and 71% respectively for patients with cyclin D1 high contra cyclin D1 moderate/low tumours (Figure 3A). In the group of >90% ER*α* positive tumours, there was an even more noticeable difference in survival for cyclin D1 high contra moderate/low tumours with mortality rates of 48 and 85% respectively (Figure 3B). This suggests that high levels of cyclin D1 were associated with an overall better prognosis than moderate or low cyclin D1 levels in untreated patients. Interestingly, the opposite was observed when analysing patients treated with tamoxifen only (Figure 3C) and especially when focusing on a rather short follow-up time. The apparent contrasting results for patients that received tamoxifen or not, clearly illustrates the importance of an untreated control patient group for prognostic studies and might further explain earlier divergent results reported concerning cyclin D1 and prognosis. The reason why the fraction of cyclin D1 and intensity of cyclin D1 produced different results regarding prognostic as well as predictive information is not clear. The fraction of cyclin D1 must be affected by the amount of cells in different cell cycle phases whereas the intensity potentially reflects the maximum protein expression independent of total proliferation. Cyclin D1 nuclear intensity has previously been linked to the degree of amplification of the cyclin D1 gene (Michalides *et al*, 1996). It is well known that high cyclin D1 protein content in tumours is not always caused by gene amplification (Buckley *et al*, 1993) and this might also influence the cyclin D1 fraction and intensity measurements in this material of breast cancer samples.

Our results using randomised untreated or tamoxifen treated patients with a long follow-up period indicate that cyclin D1 indeed affects tamoxifen response, which is in line with some of the published data using cell lines. In contrast to this study, Han *et al* (2003) did not observe any difference in tamoxifen response in metastatic breast cancer when studying cyclin D1. This study was nevertheless rather limited and the patients were not randomised, which might explain the discrepancy. The most likely mechanistic explanation for the effect of cyclin D1 on tamoxifen response is either through a direct interaction between cyclin D1 and the ER*α*/SRC-1 complex or via its cell cycle regulatory function, as also supported by cell line studies (Zwijsen *et al*, 1998). Cyclin D1 could potentially partially block the effect of tamoxifen on the ER*α* despite theoretically causing an oestrogen-independent low activation. An alternative model is that cyclin D1 could sequester cdk-inhibitors, thereby affecting the G1/S control and tamoxifen response.

**Figure 4:**
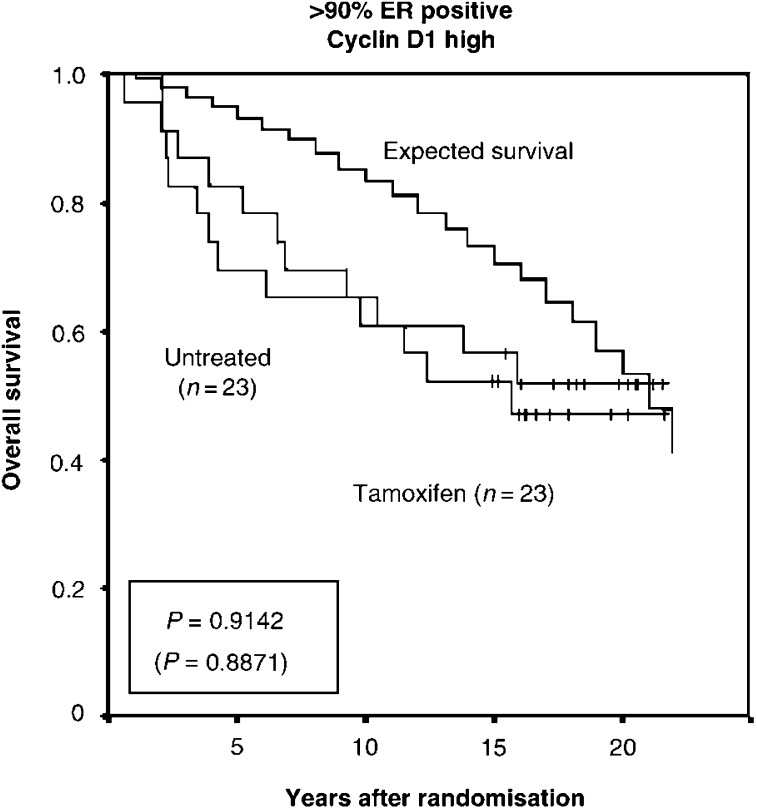
Overall survival in the patient group of >90% ER*α* positive, cyclin D1 high breast cancers subdivided according to tamoxifen treatment or no treatment with indication of a calculated expected survival for an aged-matched non-breast cancer cohort. *P*-values are presented in a box with value at 10 years of follow-up (22 years of follow-up in parenthesis).

It thus seems that a large fraction of patients who receive tamoxifen do not benefit from it and this could partially be mediated through cyclin D1. Despite the rather favourable prognosis for cyclin D1 high breast cancer patients, a substantial fraction indeed die in advance as illustrated in Figure 4

, showing the overall survival for patients with cyclin D1 high tumours compared to the expected survival in an aged-matched population. The outcome for this large cohort of breast cancer patients could nevertheless theoretically be improved by specifically targeting cyclin D1 in conjunction with tamoxifen, representing a new treatment strategy for tamoxifen resistance in ER*α* positive cyclin D1 overexpressing breast cancer.

## References

[bib1] Ali S, Coombes RC (2002) Endocrine-responsive breast cancer and strategies for combating resistance. Nat Rev Cancer 2: 101–112 12635173 10.1038/nrc721

[bib2] Bieche I, Olivi M, Nogues C, Vidaud M, Lidereau R (2002) Prognostic value of CCND1 gene status in sporadic breast tumours, as determined by real-time quantitative PCR assays. Br J Cancer 86: 580–586 11870541 10.1038/sj.bjc.6600109 PMC2375286

[bib3] Bindels EM, Lallemand F, Balkenende A, Verwoerd D, Michalides R (2002) Involvement of G1/S cyclins in estrogen-independent proliferation of estrogen receptor-positive breast cancer cells. Oncogene 21: 8158–8165 12444551 10.1038/sj.onc.1206012

[bib4] Buckley MF, Sweeney KJ, Hamilton JA, Sini RL, Manning DL, Nicholson RI, deFazio A, Watts CK, Musgrove EA, Sutherland RL (1993) Expression and amplification of cyclin genes in human breast cancer. Oncogene 8: 2127–2133 8336939

[bib5] EBCTC (1998) Tamoxifen for early breast cancer: an overview of the randomised trials. Early Breast Cancer Trialists' Collaborative Group. Lancet 351: 1451–1467 9605801

[bib6] Elwood JM, Godolphin W (1980) Oestrogen receptors in breast tumours: associations with age, menopausal status and epidemiological and clinical features in 735 patients. Br J Cancer 42: 635–644 7459204 10.1038/bjc.1980.296 PMC2010532

[bib7] Gillett C, Smith P, Gregory W, Richards M, Millis R, Peters G, Barnes D (1996) Cyclin D1 and prognosis in human breast cancer. Int J Cancer 69: 92–99 8608989 10.1002/(SICI)1097-0215(19960422)69:2<92::AID-IJC4>3.0.CO;2-Q

[bib8] Han S, Park K, Bae BN, Kim KH, Kim HJ, Kim YD, Kim HY (2003) Cyclin D1 expression and patient outcome after tamoxifen therapy in estrogen receptor positive metastatic breast cancer. Oncol Rep 10: 141–144 12469160

[bib9] Hui R, Finney GL, Carroll JS, Lee CS, Musgrove EA, Sutherland RL (2002) Constitutive overexpression of cyclin D1 but not cyclin E confers acute resistance to antiestrogens in T-47D breast cancer cells. Cancer Res 62: 6916–6923 12460907

[bib10] Hwang TS, Han HS, Hong YC, Lee HJ, Paik NS (2003) Prognostic value of combined analysis of cyclin D1 and estrogen receptor status in breast cancer patients. Pathol Int 53: 74–80 12588434 10.1046/j.1440-1827.2003.01441.x

[bib11] Kenny FS, Hui R, Musgrove EA, Gee JM, Blamey RW, Nicholson RI, Sutherland RL, Robertson JF (1999) Overexpression of cyclin D1 messenger RNA predicts for poor prognosis in estrogen receptor-positive breast cancer. Clin Cancer Res 5: 2069–2076 10473088

[bib12] Lamb J, Ladha MH, McMahon C, Sutherland RL, Ewen ME (2000) Regulation of the functional interaction between cyclin D1 and the estrogen receptor. Mol Cell Biol 20: 8667–8675 11073968 10.1128/mcb.20.23.8667-8675.2000 PMC86475

[bib13] McMahon C, Suthiphongchai T, DiRenzo J, Ewen ME (1999) P/CAF associates with cyclin D1 and potentiates its activation of the estrogen receptor. Proc Natl Acad Sci USA 96: 5382–5387 10318892 10.1073/pnas.96.10.5382 PMC21868

[bib14] Michalides R, Hageman P, van Tinteren H, Houben L, Wientjens E, Klompmaker R, Peterse J (1996) A clinicopathological study on overexpression of cyclin D1 and of p53 in a series of 248 patients with operable breast cancer. Br J Cancer 73: 728–734 8611372 10.1038/bjc.1996.128 PMC2074376

[bib15] Naidu R, Wahab NA, Yadav MM, Kutty MK (2002) Expression and amplification of cyclin D1 in primary breast carcinomas: relationship with histopathological types and clinico-pathological parameters. Oncol Rep 9: 409–416 11836618

[bib16] Neuman E, Ladha MH, Lin N, Upton TM, Miller SJ, DiRenzo J, Pestell RG, Hinds PW, Dowdy SF, Brown M, Ewen ME (1997) Cyclin D1 stimulation of estrogen receptor transcriptional activity independent of cdk4. Mol Cell Biol 17: 5338–5347 9271411 10.1128/mcb.17.9.5338 PMC232384

[bib17] Pacilio C, Germano D, Addeo R, Altucci L, Petrizzi VB, Cancemi M, Cicatiello L, Salzano S, Lallemand F, Michalides RJ, Bresciani F, Weisz A (1998) Constitutive overexpression of cyclin D1 does not prevent inhibition of hormone-responsive human breast cancer cell growth by antiestrogens. Cancer Res 58: 871–876 9500441

[bib18] Sicinski P, Donaher JL, Parker SB, Li T, Fazeli A, Gardner H, Haslam SZ, Bronson RT, Elledge SJ, Weinberg RA (1995) Cyclin D1 provides a link between development and oncogenesis in the retina and breast. Cell 82: 621–630 7664341 10.1016/0092-8674(95)90034-9

[bib19] Sorlie T, Perou CM, Tibshirani R, Aas T, Geisler S, Johnsen H, Hastie T, Eisen MB, van de Rijn M, Jeffrey SS, Thorsen T, Quist H, Matese JC, Brown PO, Botstein D, Eystein Lonning P, Borresen-Dale AL (2001) Gene expression patterns of breast carcinomas distinguish tumor subclasses with clinical implications. Proc Natl Acad Sci USA 98: 10869–10874 11553815 10.1073/pnas.191367098 PMC58566

[bib20] Thorpe SM, Christensen IJ, Rasmussen BB, Rose C (1993) Short recurrence-free survival associated with high oestrogen receptor levels in the natural history of postmenopausal, primary breast cancer. Eur J Cancer 29A: 971–977 8499151 10.1016/s0959-8049(05)80204-7

[bib21] van Diest PJ, Michalides RJ, Jannink L, van der Valk P, Peterse HL, de Jong JS, Meijer CJ, Baak JP (1997) Cyclin D1 expression in invasive breast cancer. Correlations and prognostic value. Am J Pathol 150: 705–711 9033283 PMC1858273

[bib22] Wang TC, Cardiff RD, Zukerberg L, Lees E, Arnold A, Schmidt EV (1994) Mammary hyperplasia and carcinoma in MMTV-cyclin D1 transgenic mice. Nature 369: 669–671 8208295 10.1038/369669a0

[bib23] Zhou Q, Hopp T, Fuqua SA, Steeg PS (2001) Cyclin D1 in breast premalignancy and early breast cancer: implications for prevention and treatment. Cancer Lett 162: 3–17 11121857 10.1016/s0304-3835(00)00657-1

[bib24] Zwijsen RM, Buckle RS, Hijmans EM, Loomans CJ, Bernards R (1998) Ligand-independent recruitment of steroid receptor coactivators to estrogen receptor by cyclin D1. Genes Dev 12: 3488–3498 9832502 10.1101/gad.12.22.3488 PMC317237

[bib25] Zwijsen RM, Wientjens E, Klompmaker R, van der Sman J, Bernards R, Michalides RJ (1997) CDK-independent activation of estrogen receptor by cyclin D1. Cell 88: 405–415 9039267 10.1016/s0092-8674(00)81879-6

